# Supplementary material for: Effects of storage conditions on the microbiota of fecal samples collected from dairy cattle
Source: PLoS One. 2024 Aug 9;19(8):e0308571. doi: 10.1371/journal.pone.0308571 (PMC11315314; doi:10.1371/journal.pone.0308571)
Supplement: Attachment — Submitted filename: Response to Reviewers.docx [file pone.0308571.s002.docx]

Journal: PLOS ONE

Manuscript ID: PONE-D-24-15864

Title: "Effects of storage conditions on the microbiota of fecal samples collected from dairy cattle"

Thank you for giving us the opportunity to submit a second revised draft of our manuscript titled "Effects of storage conditions on the microbiota of fecal samples collected from dairy cattle" to PLOS ONE. We appreciate the time and effort that you and the reviewers have dedicated to providing valuable feedback on our manuscript. We are grateful to the reviewers for their insightful comments on our paper. We have now been able to incorporate changes to reflect the suggestions provided by the reviewers.

**Reviewer #1**

1. I find it odd that a group of authors' who state that their expertise is not bioinformatics, would undertake to write a manuscript based almost entirely off of bioinformatics analyses, and not bring onboard an expert? It is not that the analysis was poorly done, it is just that you should not use not being an expert as an rebuttal to leave out details needed for reproducibility of the protocol.

We appreciate your comment. Although our primary expertise is not in bioinformatics, we have taken extensive measures to ensure the robustness and reproducibility of our protocol. We acknowledge the importance of detailed methodological transparency and have made every effort to include all necessary information to support reproducibility, as suggested by the reviewers.

2. For future studies, it is the responsibility of the authors to ensure that they are familiar with the workflow of the sequencing provider and that correct procedure is followed - from experience one cannot assume that providers would do the "proper" things. Surely it would have been possible to have reached out and asked the provider what kind of controls they had instead of rebutting saying you assume they did?

Thank you for your comment. We appreciate your feedback and acknowledge the importance of verifying the workflow and controls with the sequencing provider. For future studies, we will ensure to obtain detailed information directly from the provider rather than making assumptions.

3. Lastly, while the focus of the study was not to present a robust microbiota profile, I would argue that in order to properly represent the changes happening as a result of the conditions the samples were exposed to, other experimental factors such as contamination (which could skew results) should have been controlled for, and the data QC and taxonomy assignment be as robust as possible. Differential abundance is also not hard to do in QIIME2, and could have easily been applied to see which, if any, phyla are differentially abundant and therefore the main drivers of changes due tot the conditions.

As suggested by the reviewer, we have performed a differential abundance analysis, and the results are presented in the accompanying figure and table. In the differential abundance figure, the phyla Bacteroidetes and Firmicutes are differentially abundant, and their absolute abundance decreases from 0d to E conditions.

However, we chose not to include this analysis in the main text because, as noted, other experimental factors, such as contamination of the DNA, were not controlled. Thus, we cannot confidently attribute the total load changes in the two mentioned phyla's absolute abundance solely to the evaluated storage conditions. Therefore, only relative abundance data is presented in the main text because it allows for a descriptive comparison of the composition of the microbiome, regardless of the total number of bacterial reads or cells. Additionally, we improved the visualization of Figure 4, which illustrates such data.

In lines 306-309, we emphasized the importance of future studies incorporating comprehensive descriptions of bioinformatic methods and more robust DNA quality control (QC) measures. Implementing more stringent protocols in these areas will significantly enhance the validity, reliability, and reproducibility of future research findings. This includes detailed documentation of bioinformatic pipelines, standardized QC procedures to filter and normalize data, and the use of up-to-date, validated reference databases for taxonomy assignments. By adopting these practices, future studies can ensure that results are more consistent and comparable across different research settings.

4. As adding controls is clearly not possible at this stage, please at least add a statement indicating that extraction negative controls and positive controls were not included in the protocol and please consider performing more robust analyses in future. It would only serve to strengthen the results you see, regardless of the focus.

 As suggested, we have added a statement, lines in line 119 acknowledging that our protocol did not include extraction negative and positive controls. We also recognized the importance of these controls and recommended their incorporation in future studies to strengthen the robustness of the results (lines 307 – 309).

**Reviewer #2**

This reviewer did not have additional comments.
